# Impact of electronic-alerting of acute kidney injury: workgroup statements from the 15^th^ ADQI Consensus Conference

**DOI:** 10.1186/s40697-016-0101-1

**Published:** 2016-02-26

**Authors:** Eric A. J. Hoste, Kianoush Kashani, Noel Gibney, F. Perry Wilson, Claudio Ronco, Stuart L. Goldstein, John A. Kellum, Sean M. Bagshaw, on behalf of the 15 ADQI Consensus Group

**Affiliations:** Intensive Care Unit, Ghent University Hospital, Ghent University, De Pintelaan 185, 9000 Ghent, Belgium; Research Foundation-Flanders (FWO), Brussels, Belgium; Division of Pulmonary and Critical Care Medicine, Department of Medicine, Mayo Clinic, 200 First St, SW, Rochester, MN USA; Division of Nephrology and Hypertension, Department of Medicine, Mayo Clinic, 200 First St, SW, Rochester, MN USA; Division of Critical Care Medicine, Faculty of Medicine and Dentistry, University of Alberta, CSB 2-124, 8440-112 Street, Edmonton, AB Canada; Program of Applied Translational Research, Department of Medicine, Yale University School of Medicine, 60 Temple Street Suite 6C, New Haven, CT 06510 USA; Department of Nephrology and International Renal Research Institute, Ospedale San Bortolo, Vicenza, Italy; Center for Acute Care Nephrology, Cincinnati Children’s Hospital Medical Center, 3333 Burnet Avenue, MLC 7022, Cincinnati, OH 45229 USA; Center for Critical Care Nephrology, Department of Critical Care Medicine, University of Pittsburgh, Pittsburgh, PA USA; Division of Critical Care Medicine, Faculty of Medicine and Dentistry, University of Alberta, 8440-112 ST NW, Edmonton, AB T6G2B7 Canada

**Keywords:** Acute kidney injury, Sniffer, Electronic alert, Electronic health records

## Abstract

**Purpose of the review:**

Among hospitalized patients, acute kidney injury is common and associated with significant morbidity and risk for mortality. The use of electronic health records (EHR) for prediction and detection of this important clinical syndrome has grown in the past decade. The steering committee of the 15^th^ Acute Dialysis Quality Initiative (ADQI) conference dedicated a workgroup with the task of identifying elements that may impact the course of events following Acute Kidney Injury (AKI) e-alert.

**Sources of information:**

Following an extensive, non-systematic literature search, we used a modified Delphi process to reach consensus regarding several aspects of the utilization of AKI e-alerts.

**Findings:**

Topics discussed in this workgroup included progress in evidence base practices, the characteristics of an optimal e-alert, the measures of efficacy and effectiveness, and finally what responses would be considered best practices following AKI e-alerts. Authors concluded that the current evidence for e-alert system efficacy, although growing, remains insufficient. Technology and human-related factors were found to be crucial elements of any future investigation or implementation of such tools. The group also concluded that implementation of such systems should not be done without a vigorous plan to evaluate the efficacy and effectiveness of e-alerts. Efficacy and effectiveness of e-alerts should be measured by context-specific process and patient outcomes. Finally, the group made several suggestions regarding the clinical decision support that should be considered following successful e-alert implementation.

**Limitations:**

This paper reflects the findings of a non-systematic review and expert opinion.

**Implications:**

We recommend implementation of the findings of this workgroup report for use of AKI e-alerts.

## ABRÉGÉ

**Contexte et objectifs de la revue:**

L’insuffisance rénale aigüe (IRA) est un problème de santé fréquent chez les patients hospitalisés, et elle présente un risque élevé de morbidité et de mortalité pour les personnes affectées. L’utilisation des dossiers médicaux électroniques (DMÉ) pour la prédiction et le dépistage de ce syndrome clinique est en croissance depuis une dizaine d’années. Le comité directeur de la 15^e^ réunion annuelle de la *Acute DIalysis Quality Initiative* (ADQI) a désigné un groupe de travail à qui il a donné le mandat d’identifier les éléments susceptibles d’avoir une incidence sur le cours des événements à la suite d’une alerte électronique indiquant un changement dans le taux de créatinine sérique d’un patient (alerte électronique d’IRA).

**Sources et méthodologie:**

À la suite d’une revue exhaustive, mais non systématique de la littérature, nous avons utilisé une version modifiée de la méthode Delphi afin de parvenir à un consensus sur plusieurs facteurs liés à l’utilisation des alertes électroniques IRA.

**Résultats/constatations:**

Parmi les thèmes discutés par ce groupe de travail figuraient les progrès observés au niveau de la pratique factuelle, l’identification des caractéristiques d’une alerte électronique optimale, la façon de mesurer l’efficacité des alertes et enfin, les interventions qualifiées de pratiques exemplaires à appliquer à la suite d’une alerte électronique d’IRA. Les auteurs ont conclu que les connaissances actuelles sur l’efficacité des systèmes d’alertes électroniques, bien qu’en progression, demeurent insuffisantes. Ils ont de plus identifié les facteurs humains et technologiques comme étant des éléments clés à considérer lors d’investigations futures portant sur de tels systèmes ou lors de leur mise en œuvre dans le futur. Le groupe de travail a également conclu que la mise en place de tels systèmes d’alertes ne devrait toutefois pas se faire sans un programme rigoureux d’analyse de l’efficacité et de l’efficience des alertes émises, et que ces mesures devraient se faire dans un cadre précis et en tenant compte des résultats observés chez les patients. Enfin, les auteurs ont fait plusieurs suggestions de mécanismes d’aide à la prise de décisions cliniques à prendre en considération à la suite de la mise en œuvre réussie d’un système d’alertes électroniques.

**Limites:**

Cet article fait état des conclusions obtenues dans le cadre d’une revue non systématique de la littérature et à partir des opinions d’un groupe d’experts.

**Conclusion:**

Nous recommandons la mise en application des conclusions émises dans le rapport présenté par le groupe de travail sur l’utilisation des alertes électroniques IRA.

## Background

Acute kidney injury (AKI) is defined by the Kidney Disease: Improving Global Outcomes (KDIGO) definition, which is a modification of the RIFLE (risk, injury, failure, loss, and end-stage kidney disease) and acute kidney injury network (AKIN) consensus definitions for AKI [1–3]. This definition involves the evaluation of an absolute or relative increase in serum creatinine (hereafter called 'creatinine') or oliguria for six or more hours. At first sight, these criteria seem simple and straightforward. However, appropriate detection of AKI requires knowledge of a baseline creatinine or reference creatinine, calculation of urine output / body weight per hour, and calculation of time periods during which the change in creatinine or urine output occurs [4]. This makes an evaluation of the occurrence of AKI and staging of severity complex and labor-intensive.

Information technology is increasingly used in the healthcare setting for the integration of all available data as an aid to clinical decision-making. The individual elements that are necessary for the definition and staging of AKI are typically available in the integrated electronic health record (EHR) or intensive care clinical information system. Therefore, an electronic sniffer or electronic-alert (e-alert) can potentially detect AKI each time creatinine or urine output is recorded.

The steering committee of the 15^th^ Acute Dialysis Quality Initiative (ADQI) conference dedicated a workgroup with the task of considering elements that may impact the course of events following AKI e-alert. More specifically, they were asked to address a set of 4 questions:

1. What is the evidence base regarding AKI e-alerting?
2. What are the characteristics of an optimal e-alert?
3. How should we assess the efficacy and effectiveness of e-alerts?
4. What responses can be considered best practices?

These questions served as a basis for accompanying consensus statements. Our group was also asked to provide a critical evaluation of the relevant literature to summarize the methodology, scope, implementation and evaluative strategies for EHR-based clinical decision support.

### Review

This consensus meeting following the established ADQI process, as previously described [5]. The broad objective of ADQI is to provide expert-based statements and interpretation of current knowledge for use by clinicians according to professional judgment and identify evidence care gaps to establish research priorities.

The 15th ADQI Consensus Conference Chairs convened a diverse panel representing relevant disciplines (i.e., nephrology, critical care, pediatrics, pharmacy, epidemiology, health services research, biostatistics, bioinformatics and data analytics) from five countries from North America and Europe around the theme of “Acute Kidney Injury in the Era of Big Data” for a 2-day consensus conference in Banff, Canada on September 6-8, 2015.

Before the conference we searched the literature for evidence on methodologies for design, integration and implementation of novel applications into the electronic health records that enable "alerting" of changes in clinical status and provide a modality of clinical decision support. A formal systematic review was not conducted.

A pre-conference series of call conferences and emails involving the work group members was used to identify the current state of knowledge to enable the formulation of key questions from which discussion and consensus would be developed.

During the conference, our work group developed consensus positions, and plenary sessions involving all ADQI contributors were used to present, debate, and refine these positions.

Following the conference, this summary report was generated, revised, and approved by all members of the workgroup.

### What is the evidence base regarding AKI e-alerting?

#### Consensus statement 1

Current evidence is limited by the number of studies, their heterogeneity (design of the sniffer, location, clinical action, outcomes measured, etc.), and contradictory results.

An overview of studies that report on the use of e-alerts for AKI is presented in Table 1. We identified two groups of studies on e-alerts and AKI. The first category reported the utilization of an e-alert without measurement of their impact on the process of care and patient or kidney outcomes [6–12]. In the second group, processes of care or outcomes were measured, but e-alerting did not improve outcomes [13–15]. Finally, in the third set of studies, recorded clinical outcomes or quality of care indicated improvement [16–26]. Despite a relatively large number of patients studied, the actual number of centers where these e-alerts were evaluated was limited. In addition, we found that there was considerable heterogeneity among studies, which makes systematic analysis difficult.

**Table 1:** Use of electronic alerts for detection of acute kidney injury in clinical studies

Study	Number of participants	Setting	Process of care	Outcome
Studies reporting on the use of e-alerts without measurement of process of care or outcome				
Colpaert (2007) [6]		ICU		
Thomas (2011) [7]	463 patients	2 hospitals		
Selby (2012) [8]	2619 patients	1 hospital		
Porter (2014) [9]	15,550 patients/22,754 admissions	2 hospitals		
Handler (2014) [10]	249 patients	4 nursing homes		
Wallace (2014) [11]	23,809	Hospital		
Ahmed (2015) [12]	944	ICU		
Studies reporting on the use of e-alerts: no improvement reported				
Sellier (2009) [13]	603	Hospital	No impact on prescription errors	
Thomas (2015) [14]	308	Hospital		No difference in outcome of AKI
Wilson (2015) [15]	23,664	Hospital		No effect on AKI rate
Studies reporting on the use of e-alerts: improvement reported				
Rind (1991) [16]	10,076 patients /13,703 admissions	Hospital	Adjustment of medication sooner	
Rind (1994) [17]	20,228 admissions	Hospital	Adjustment of medication sooner	Decreased risk for AKI
Chertow (2001) [18]	17,828 patients	Hospital	More adequate antibiotic prescription	
McCoy (2010) [19]	1237 patients	Hospital	More adequate medication prescription	
Terrel (2010) [20]	2783 patients visits	Emergency room	More adequate dosing	
Cho (2012) [21]	463 patients	Hospital	More contrast prophylaxis	Less AKI
Colpaert (2012) [22]	951 patients	ICU	More and earlier interventions for AKI	Less progression AKI
Goldstein (2013) [23]	21,807 patients/27,711 admissions	Pediatric hospital		Less AKI
Selby (2013) [24]	8411 patients	Hospital		Decreased mortality AKI
Claus (2015) [25]	87 patients	ICU	Decrease workload pharmacist	
Kolhe (2015) [26]	2297 patients	Hospital		Less AKI progression Decreased mortality

### What is an optimal e-alert?

#### Consensus statement 2

There are several technological and human factors that need to be considered during the implementation and evaluation of an AKI e-alert system. These elements include but are not limited to the clinical context, location, provider, e-alert accuracy, the hierarchy of disruptiveness (i.e., the extent to which the alert disrupts the current workflow), delivery methods, alarm philosophy, and outcome expectations in clinical and administrative settings.

**Fig. 1:**
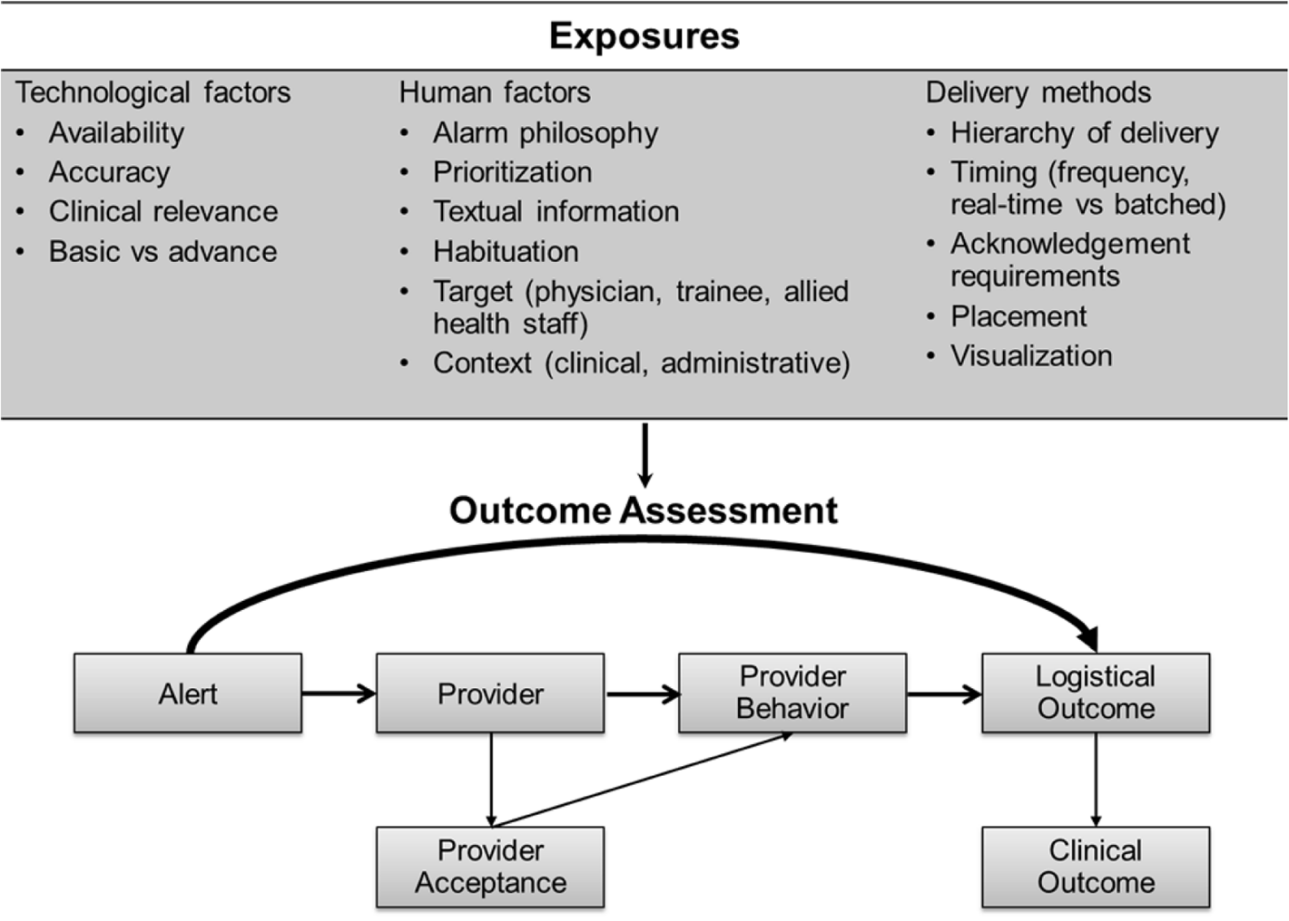
The process of electronic-alert from exposure to outcome. An e-alert should impact on logistical or clinical outcomes. In this process exposure to the e-alert components (technology and human factors, delivery methods) potentially results in a change of behavior of the provider. Crucial to this process is the acceptance of the alert by the provider. Reproduced with permission from ADQI (www.adqi.org)

The turn of events that leads to the process of care modification or clinical outcomes following the firing of an e-alert is illustrated in Fig. 1. Although the role of the EHR in the care and management of patients with AKI is potentially important, literature regarding the characteristics of an effective AKI e-alert is scarce. Several components have been described to change the effectiveness and acceptance of e-alert systems for other clinical and administrative purposes. The depth of knowledge that is generated by the EHR could be divided to basic and advanced. Basic e-alerts disregard the clinical context or have low precision; therefore, it is not surprising that e-alerts with basic capabilities are not widely accepted in the clinical practice [27–36]. In comparison, advanced e-alerts assist clinicians by including information regarding the clinical context and possess significantly higher sensitivity and specificity. Advanced e-alerts can potentially play a significant role in easing the heavy workload of clinicians by enhancing safety measures and efficacy without creating a distraction.

Despite the advantages of utilizing the e-alert system abilities, the method of delivery may impact their acceptance into the clinical practice. Phansalkar et al. described these features as human factors and divided them into several distinct elements [37, 38]. These components include: alarm philosophy (defining the unsafe situations that require alarming), placement (within or outside of visual horizon), visualization (target size, luminance, background contrast), prioritization (using appropriate wording for different urgency levels), textual information (to include priority, information regarding the nature of the alert, recommendation and a statement to indicate the consequence of ignoring the alert), and habituation (decreased response to alarms over time). Implementation of irrelevant alarms also has an adverse impact on the acceptance of e-alerts by clinicians. These type of alarms could be defined as warnings that do not require a response by care providers. They are irrelevant to the patient quality of care and safety, or they generate significant false positive warnings. Further, Seidling et al. included these factors in a scale and based on their performance and characteristics divided them to poor, moderate and excellent e-alerts [39]. In order to set up a successful e-alert system, one needs to consider other variables including the patient setting (intensive care units (ICU) where patients already undergo close monitoring, versus hospital ward or outpatient clinic in which patient data are scarce), the hierarchy of disruption (the spectrum of disruption ranging from no-alert to a hard stop without rights to override), frequency of alerts (alert submission till resolution of the issue versus alert submission only once), timing (real time versus set time for submission all in clusters), provider acknowledgement requirements (no need for response versus punitive measures if response is not provided), target of e-alert (physician, midlevel provider, trainees, nurses, or patients), and finally content of alarm (AKI diagnosis or risk prediction, and clinical decision support). Furthermore, cultural differences based on the type (community versus teaching) and size (small versus large hospitals) of the institution, geographical locations (continents, countries, counties), services (medical versus surgical), providers (subspecialists, specialists, midlevel, trainee, allied health staff) could significantly impact the performance of e-alerts in improvement of patient care and safety. Finally, what is expected from the e-alert system may define its success or failure. For example, if the expectation is to improve mortality of hospitalized patients, the alerts need to be very precise, disruptive, be tagged with a very sophisticated clinical decision support system, and if any study aims to show its efficacy it needs to include a very large number of patients. In comparison, when e-alerts are used for administrative purposes the level of disruptiveness and their required precision could be completely different.

To provide an example of how differences in the aforementioned factors can affect the performance of AKI e-alerts in various platforms, we present two recent published studies that focused on the impact of AKI e-alerts on patient and processes of care outcomes. Colpaert et al. described a single-center European prospective interventional study in which she used AKI alert via Digital Enhanced Cordless Technology (DECT) telephone to the intensivists [22]. This alert included information regarding changes in creatinine and urine output, and the alert was generated whenever AKI progressed to the next stage of RIFLE criteria [2]. She compared the processes of care in the periods before, during, and following alert implementation and found a significant increase in the number and timeliness of early therapeutic interventions during the alert phase. In comparison, Wilson et al. recently published results of a randomized controlled trial to evaluate the impact of a single alert via pager on the outcomes of hospitalized patients in a single center in the United States [15]. Alerts were generated solely based on absolute or relative rise in the creatinine level in comparison with the lowest creatinine level measured within the past 48 h (for 26 mmol/L [0.3 mg/dL] criteria) or 7 days (for 50 % relative increase criteria). Authors included adult patients from the medical and surgical ICUs, and floors and providers who received alerts were interns, residents, or nurse practitioners. This study did not show any improvement in the clinical outcomes or processes of care among hospitalized patients. These contrasting results highlight the importance of the e-alert system design and human factors on the clinical performance of the system.

### How do we measure alert efficacy?

#### Consensus statement 3

E-alert efficacy and effectiveness should be measured proactively and encompass quality assurance, provider-based responses, and clinical outcomes.

The use of e-alerts for a variety of conditions has expanded dramatically in the past several years but has also placed new burdens on providers [38, 40–43]. In the best case scenarios, alerts can prevent medical error or promote timely and appropriate treatment of a severe condition. In the worst case scenarios, they can impede workflow, distract providers, and lead (indirectly) to patient harm.

Therefore, e-alert systems should not be adopted without a rigorous assessment of their benefit and risk across several domains. These evaluations, where possible, should be performed in the context of a randomized, controlled trial. However, even in settings where the performance of a randomized trial is not feasible, attention to key metrics both before and after e-alert implementation will aid in the assessment of efficacy.

Prior to the wide rollout of e-alert systems for AKI, careful testing of the system should be performed. Pre-testing of the system should include a systematic effort to determine whether the e-alert is capturing all patients of interest (by whatever AKI definition is being used) and is not erroneously alerting for patients without AKI. This may be a particular issue for individuals receiving dialysis for end-stage kidney disease, in whom inter-dialytic creatinine fluctuation may trigger alerts. In addition, a recent study has shown that false-positive AKI rates may be particularly high among individuals with chronic kidney disease when electronic monitoring of creatinine levels is employed [44].

After the alert system is appropriately calibrated, developers must ensure that the proper alert target is identified and reached. Challenges here can include identifying who the appropriate care provider or providers are to receive an alert and the mechanism by which they can be contacted.

Developers of e-alerts should perform system-wide implementation only when the above measures have been satisfied. Once alerting is broadly executed, several other efficacy metrics become important.

Depending on the context of the e-alert, various provider behaviors should be evaluated. Broadly, we consider provider-initiated electronic documentation of AKI and orders for follow-up creatinine and urine output assessment to be important metrics of alert efficacy. Other provider behaviors (such as the ordering of certain diagnostic tests, studies, changing drug dosing, and avoidance of nephrotoxins) may be appropriate efficacy measures in certain clinical contexts.

Provider actions, such as ordering subsequent lab testing, should be examined independently of the successful completion of the order (the actual blood being drawn). This ensures robust assessment of efficacy as well as avoiding systematic "workarounds". For example, if a provider is aware that an order for another creatinine test is a quality measure, he or she may order the test with no intention of having the test performed (for example, by specifying the blood to be drawn at a time after the patient is to be discharged).

Critically, clinical outcomes should be assessed in all e-alert systems, as there is some evidence that e-alerts may increase resource utilization without tangible patient-level benefit [15]. In the case of AKI e-alerts, clinical outcomes can include the receipt of dialysis, death, ICU transfer, and change in creatinine concentration among others.

We also suggest that efforts be made to gauge provider acceptance of e-alert systems. These studies can be quantitative or qualitative, but they should be undertaken concurrently with e-alert development and with the understanding that e-alert systems that do not integrate well into a provider perception of care are unlikely to demonstrate sustained benefit.

### What responses can be considered best practices?

#### Consensus statement 4

Following AKI alert (risk or diagnosis), the clinician should confirm and document the risk or diagnosis in the clinical notes and EHR. Follow-up measurement of urine output and creatinine should be ordered, and the use of additional diagnostics be considered. Appropriate care or recommendations according to the best evidence-based practices for prevention or treatment should be utilized, and the effectiveness of clinical decision support systems (CDS) should be evaluated.

Increased severity of AKI is associated with increasing risk of death and other serious complications [45]. Therefore, there is increasing focus on the importance of early recognition and management of AKI, with the goal of potentially providing a wide therapeutic window for prevention and treatment [1, 46]. The use of e-alerts to enhance the compliance with AKI-related clinical practice guidelines offer the potential for minimizing the impact of AKI [1, 22, 26, 27]. However, it is evident that physician notification by e-alert alone is not adequate to ensure an optimal response in patients with probable AKI [15]. Alerts should be combined with clear institutional clinical practice guidelines or care bundles outlining the most appropriate response to the level of the e-alert.

A number of clinical audits of patients with AKI have identified deficiencies in the identification, documentation and intervention [47, 48]. Among others, these include failure to diagnose and document AKI, to adequately assess the patient’s clinical status or to measure urine output and sequential creatinine levels and withhold or dose-adjust nephrotoxic medications.

Comprehensive clinical practice guidelines have been developed by KDIGO, the UK National Clinical Guideline Centre, and other groups, for the recognition and management of patients with AKI [1, 49]. In addition, some healthcare centers have developed AKI care checklists to facilitate early recognition and appropriate management of patients with AKI [26, 50]. Tsui et al. devised an AKI care bundle to guide the clinical response in patients with AKI [50]. The impact of implementing an AKI care bundle was studied in patients with new-onset AKI. This involved a hospital-wide education campaign, although an e-alert system was not used. Improved compliance with appropriate investigations and initial treatments was associated with a decreased requirement for ICU admission and a trend towards a shorter length of stay.

Kohle et al. developed an AKI care bundle and combined this with an e-alert system to notify physicians that their patients may have developed AKI [26]. Outcomes were compared in patients who had the care bundle completed within 24 h of AKI alert versus those who did not. Progression to higher AKI stages was lower in patients in whom the care bundle was implemented within 8 h. These patients also had lower odds of death at discharge and up to 4 months post discharge.

Despite the development of guidelines for the staging and classification of AKI and chronic kidney disease (CKD), kidney disease is poorly documented in physician notes, suggesting both lack of recognition and understanding of the importance of documentation for diagnostic coding in administrative databases and institutional reimbursement [51, 52]. Consequently, following receipt of an AKI e-alert and assessment of the patient, the physician who has been notified should document the presence of the appropriate AKI stage in the patient’s file, problem list, and EHR. Consideration should be given to the automatic exportation of this data to the institutional administrative, and diagnostic coding system.

The minimal clinical response to an e-alert suggesting the presence or risk of AKI should be a full clinical and laboratory reassessment of the patient as well as a review of all medications by the provider receiving the e-alert.

Following the appropriate design of e-alert systems, effective utilization of change management tools and educating all stakeholders determines the success of e-alerts. In the first step, awareness of the need to such e-alert systems needs to be raised. Investigators and clinicians must conduct studies to show improvement in the processes of care or patient clinical outcomes, utilizing such systems. In this stage, communication with all stakeholders and asking them for their input is essential. In the next phase, desire to participate and support using these tools need to be instigated among all clinicians and care providers. Providing incentives to the e-alert targets would increase the chance of e-alert implementation success. Some of these incentives are an enhancement in patient safety and quality of care, easing the information overload and increasing hospital income by appropriate documentation. Following raising awareness and creating a desire to participate, stakeholders need to be educated on the use of e-alerts and clinical decision support systems. In this phase, some examples of best practices could be catered to the clinicians to be used as role models. Constant coaching and mentoring, and removing the bottlenecks are steps need to be taken to the next stage. And finally, e-alert utilization should be reinforced by continuous supplement of appropriate information regarding improvement in patient outcomes or hospital reimbursement, and even enhanced physician reputation. Care providers then are encouraged to improve their effort in the implementation of e-alert systems. Change management tools like ADKAR (Awareness, Desire, Knowledge, Ability, and Reinforcement) should be considered for successful implementation of a well-designed and targeted e-alert [53].

## Conclusion

The current evidence for e-alert system efficacy and effectiveness, although growing, remains insufficient. Technology-related and human factors are crucial elements of any future investigation or implementation of such tools. Implementation of such systems should not be done without a vigorous plan to evaluate the efficacy and effectiveness of e-alerts. Efficacy and effectiveness of e-alerts should be measured by context-specific process and logistical outcomes. The evidence-based clinical decision support that should be considered following successful e-alert implementation include but not limited to appropriate documentation of AKI, ordering context specific tests, evaluation of etiology and providing context specific management and therapeutic options.

## Abbreviations

ADQI: Acute Dialysis Quality Initiative
AKI: Acute Kidney Injury
AKIN: Acute Kidney Injury Network
CDS: Clinical Decision Support
CKD: Chronic Kidney Disease
e-alert: electronic-alert
KDIGO: Kidney Disease: Improving Global Outcomes
RIFLE: Risk, Injury, Failure, Loss, End-stage renal disease definition for AKI
